# Reproductive and Exogenous Hormone Factors in Relation to Risk of Meningioma in Women: A Meta-Analysis

**DOI:** 10.1371/journal.pone.0083261

**Published:** 2013-12-27

**Authors:** Zhen-Yu Qi, Chuan Shao, Yu-Lun Huang, Guo-Zhen Hui, You-Xin Zhou, Zhong Wang

**Affiliations:** Department of Neurosurgery, The First Affiliated Hospital of Soochow University, Suzhou, Jiangsu, China; Geisel School of Medicine at Dartmouth College, United States of America

## Abstract

**Background and Objective:**

A number of studies have focused on the association between oral contraceptive (OC), hormonal replacement therapy (HRT) and reproductive factors and meningioma risk, but the results were inconsistent. Thus, a meta-analysis was performed to obtain more precise estimates of risk.

**Methods:**

We conducted a literature search using PubMed and EMBASE databases to July2013, without any limitations. Random effects models were used to summarize results.

**Results:**

Twelve case-control and six cohort studies were included in this meta-analysis. We found that an increased risk of meningioma was associated with HRT use(RR = 1.19, 95% CI = 1.01–1.40), postmenopausal women(RR = 1.32, 95% CI = 1.07–1.64) and parity(RR = 1.18, 95% CI = 1.00–1.40).No significant associations were observed for OC use (RR = 0.93, 95% CI = 0.83–1.03), age at menarche(RR = 1.06, 95% CI = 0.92–1.21), age at menopause(RR = 1.03, 95% CI = 0.81–1.30), or age at first birth(RR = 0.94, 95% CI = 0.80–1.10).

**Conclusion:**

In conclusion, the results of our study support the hypothesis that longer exposure to effect of female sex hormones may increase the risk of meningioma in women, yet additional studies are warranted to confirm our findings and identify the underlying biological mechanisms.

## Introduction

Meningiomas are largely benign tumors, which arise from meningothelial cells of the arachnoid membrane and usually have an extended duration of asymptomatic subclinical disease before presentation to medical attention [1]. The majority of meningiomas are intracranial (around 90%); spinal meningiomas account for about 10% of all meningiomas [2]. Despite decades of research, very little is known about the etiology of meningiomas. The only well-established risk factors, including ionizing radiation and certain rare genetic syndromes, can explain a small proportion of cases [3], [4]. Several other risk factors such as smoking, mobile phone use, head trauma, asthma and occupational exposures, have been suggested as potential risk factors, but the evidence is inconsistent or no definitive conclusion has been drawn [3]–[5].

The incidence of meningioma is about 2 fold higher in women than in men, which implies sex hormones could influence the development and growth of meningioma [5]. Molecular studies have shown progesterone and estrogen receptors are expressed in meningioma in various degrees [6], [7], and progesterone and estrogens together could stimulate the meningioma cells proliferation [8]. Furthermore, some clinical studies reinforced the molecular data: meningioma increases the tumor growth rate during the reproductive life period [9], [10], and an association between meningioma and breast cancer has been reported [11], [12].In recent years, numerous studies have assessed the relationship between meningioma risk and OC, HRT and reproductive factors [13]–[28].However, the results obtained so far were inconsistent and inconclusive. Therefore, a meta-analysis was performed to quantify the effect of OC, HRT and reproductive factors on meningioma incidence.

## Methods

### Publication Search

PubMed and EMBASE databases were searched with the terms**“**(“meningioma” OR “brain cancer” OR “brain neoplasms” OR “brain tumor”) AND (“reproductive factors” OR “menstrual factors” OR “age at menarche” OR “menarche” OR “menstruation” OR “parity” OR “gravidity” OR “pregnancy” OR “breastfeeding” OR “miscarriage” OR “abortion” OR “fertility” OR “age at first birth” OR “age at menopause” OR “menopausal status” OR “climacteric” OR “reproductive history” OR “estrogens” OR “sex hormones” OR “ovariectomy” OR “oophorectomy” OR “hysterectomy” OR “sex differences” OR “hormone” OR “exogenous hormones” OR “exogenous hormones use” OR “oral contraceptives” OR “hormone replacement therapy” OR “menopausal hormone therapy”) AND (“risk assessment” OR “risk” OR “risk factors”)**”**. No restrictions on language or date of publications were imposed. Searches were conducted independently by two reviewers (ZYQ and CS), and the latest search was performed on July 17, 2013.The reference lists of identified articles were also screened for additional studies.

### Inclusion criteria

We included studies that met the following inclusion criteria: (1) have cohort or case–control study design; (2) assess the association between OC, HRT, and reproductive factors and meningioma risk; (3) provided ratio (OR),relative risk (RR), or hazard ratio (HR) with corresponding 95% CIs or sufficient data to calculate them; (4) in case of multiple reports of the same study, we selected the most recent publication with the largest number of subjects; (5) we excluded the studies which involved total brain tumors or central nervous system (CNS) tumors, since total brain tumors or CNS tumors contain other types of tumors which are very different from meningioma in a pathological and clinical point of view.

### Data extraction

Two authors (ZYQ and CS) independently extracted the following data from each available study: the first author's last name, publication date, country in which performed, study period/follow-up year, age of subjects, study design, number of cases/controls (cohort), methods of data collection and matching or adjustments. Any discrepancies were resolved by discussion.

### Statistical analysis

In this meta-analysis, a case-control study nested in a cohort study was considered to be a case-control study. The RR was used as the measure of association across studies. HRs and ORs were directly considered as RRs. We extracted the risk estimates that were adjusted for the greatest number of potential confounders; however, when unavailable, unadjusted RRs were included. The unadjusted RRs were extracted directly from the article or computed from the exposure distributions for cases and controls given in the papers. We used the random rather than fixed-effects model to estimate pooled RRs because in the absence of heterogeneity, the random-effects model exactly equals the fixed-effects model and the results from random-effects model are more conservative [29].Heterogeneity across studies was evaluated by the Q statistic and considered significant when P<0.1[30].We also calculated the I^2^statistic, which is a quantitative measure of inconsistency across studies. The I^2^ statistic takes values ranging from 0 to 100% and I^2^>50% is considered to be indicative of heterogeneity [31].When significant heterogeneity was observed, subgroup analyses were performed according to study design(prospective vs. retrospective) and geographic regions (North America vs. Europe). Potential publication bias was assessed by Egger's regression test and P<0.05 was considered indicative of significant publication bias [32].

Combined risk estimates were calculated for exposure variables that were provided in at least five studies, which included OC, HRT, age at menarche, age at menopause, menopausal status, parity (numbers of live births or full-term pregnancies), and age at first birth. For OC and HRT, the most common definition of exposure among the included studies was “ever use versus never use”. Therefore, this was chosen to be the focus of the main analysis. Seven studies did not provide results for ever versus never use of HRT or OC, but provided the exposure distributions for cases and controls [13], [16], [19], [21], [25], [27], [28]. We calculated the unadjusted risk estimates and used them in our meta-analysis. Concerning reproductive factors, we performed a meta-analysis of the comparison of the highest versus lowest category in each study. For menopausal status, three unadjusted risk estimates[13], [25], [28] were computed and used in our study due to the following reasons: one study used postmenopausal women as the reference group [13], whereas six studies used premenopausal women as the reference group [15], [17]–[19], [25], [28]; the other two studies provided stratified results [25], [28]. For parity (numbers of live births or full-term pregnancies), one study in which parity was defined as the number of pregnancies lasting 6 months or longer [16], was also included in this meta-analysis. Sensitivity analyses were performed to investigate the influence of a single study on the overall risk estimate by excluding one study in each turn. In addition, we conducted an alternative sensitivity analysis which excluded studies that did not adjust for any confounders.

All statistical analyses were conducted with the STATA software, version 11.0 (STATA Corporation, College Station, TX, USA).

## Results

### Literature search

**Figure 1:**
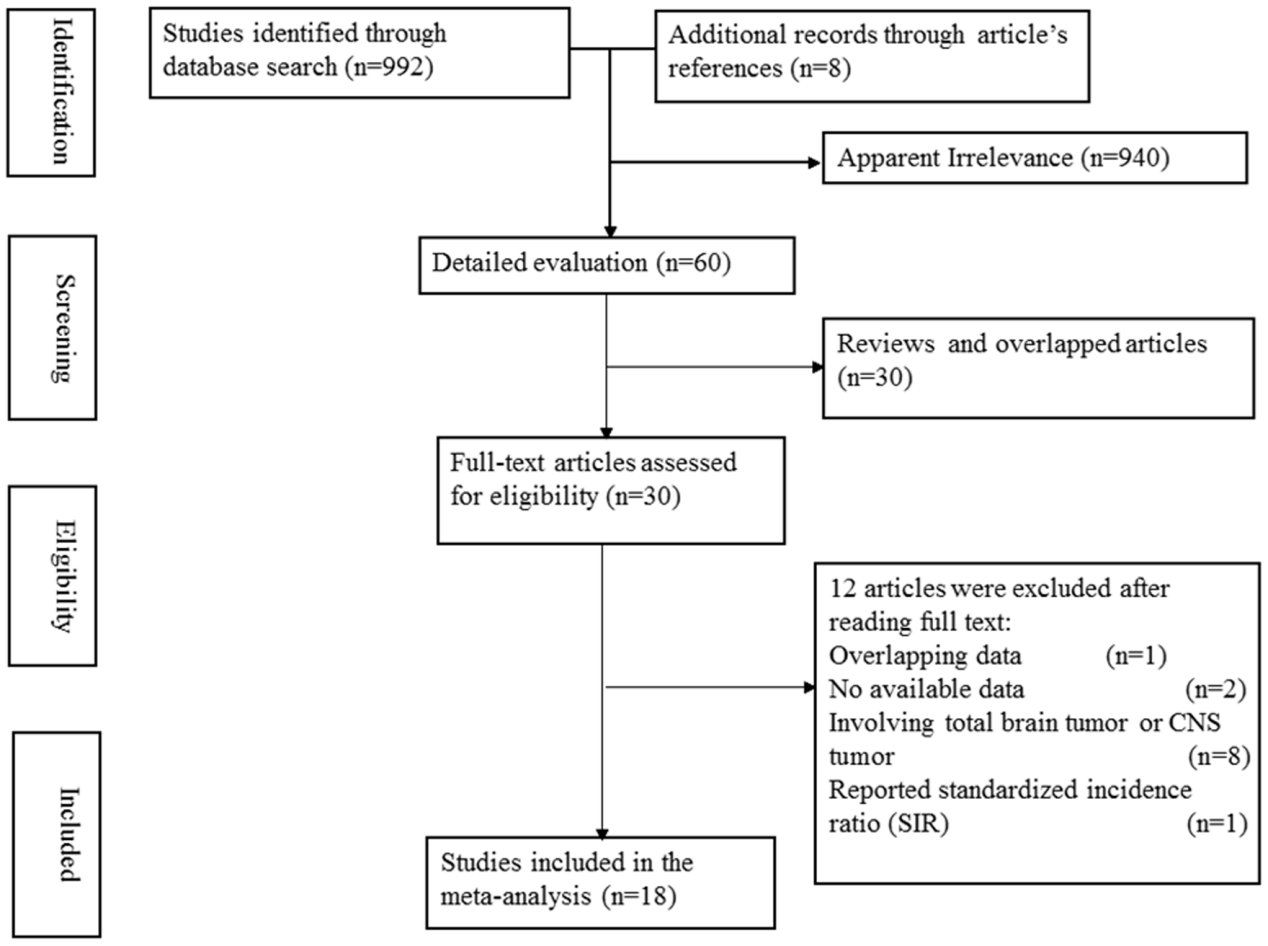
Flow diagram of literature search and selection.

We initially identified 992 potentially eligible studies (416 from PubMed, 576 from EMBASE).8 articles which may be related to the topic were found in article reference lists. Of these1000 studies, 30 records with full text that met the inclusion criteria were assessed. After reading the full-text articles, 12 studies were excluded for the following reasons: two articles have some partially overlapping data [15], [34], and the most recent article was included [15]; two articles did not have available data [35], [36]; eight articles investigated total brain tumor or CNS tumors as subjects [37]–[44]; and one article reported the standardized incidence ratio of meningioma in women who had used postmenopausal hormone therapy [45]. Thus, a final total of 18 studies published from 1995 to 2013 were included in this meta-analysis [13]–[28], [33], [46]. The flow diagram for literature search and selection of articles is presented in Figure 1.

### Study characteristics

All studies were published in English. Most of the articles were case-control studies [13]–[15], [17]–[20], [22], [24], [27], [28], [46], whereas six were cohort studies [16], [21], [23], [25], [26], [33]. Studies were conducted in Western countries, which included USA, Australia, Sweden, France, Canada, Finland, Denmark, Germany, Norway, Spain, Italy, Greece, the Netherlands, and the United Kingdom. Of 18 studies, eight studies concerned spinal and intracranial meningioma [14]–[16], , while one only investigated spinal meningioma [13], and nine only involved intracranial meningioma [17], [19], [20], [22], [24], [25], [28], [33], [46].The vast majority of cases were histologically confirmed [13]–[28], [33], [46]. However, in three studies, case definition was on the basis of radiological images for some cases [20], [22], [33].In Jhawar and colleagues' study, the definition of some cases was based on subject self-report [16]. Data were collected by questionnaire, phone interview, in person interview, or reviewing medical records. The additional characteristics of the included studies are presented in Table 1.

**Table 1:** Characteristic of the included studies in this meta-analysis.

First author, Publication date	Country^a^	Study period/Follow-up(years)	Age(years)	Study design	Cases/controls or cohort	Methods of data collection	Matching or adjustment	Exposures variables
Preston-Martin, 1995	1	1978-1985	20–74	PCC	81/155	Telephone interview	Age	HRT, OC, menopausal status, age at menarche, age at menopause.
Lambe, 1997	2	1958–1990	>15	NCC	1088/5440	Data recorded in Fertility Registry and the Swedish Cancer Registry	Age	Age at first birth, parity.
Schlehofer, 1999	1,2,7,8,9,12	1980–1991	20–80	PCC	237/637	Self-administered questionnaire or in-person interview	Age, ethnicity, residential area	Menopausal status,
Hatch, 2005	1	1994–1998	≥18	HCC	151/436	In-person interview	Age, ethnicity, hospital, residential area, marital status, and education	HRT, OC, menopausal status, age at menarche, age at menopause, age at first birth, parity.
Custer, 2006	1	1995–1998	≥18	PCC	143/286	In-person interview	Age, education, smoking history, alcohol consumption, BMI, age at menarche, and parity.	HRT, OC, menopausal status, age at menarche, age at first birth, parity.
Lee, 2006	1	1987–1992	Not Stated	HCC	219/260	Self-mailed questionnaire	Age, ethnicity, hospital, smoking, pregnancy, thyroid disorders, radiation treatment, menopause,	HRT, OC, menopausal status, age at menarche.
Wigertz, 2006	2	2000–2002	20–69	PCC	178/323	In-person interview or phone interview, Self-administered questionnaire	Age, residential area, education, and parity.	HRT, OC,
Wigertz, 2008	2,3,4,5,6	2000–2004	18–69	PCC	906/1774	In-person interview or phone interview	Age, country, residential area, and education	Age at menarche, age at menopause, age at first birth, parity.
Korhonen, 2010	4	2000–2002	20–69	PCC	264/505	In-person interview	Age, residential area, family history with brain tumors	HRT, OC, age at menarche, parity.
Claus, 2013	1	2006–2011	29–79	PCC	1127/1109	Telephone interview	Age, race, education, smoking, alcohol use, BMI	HRT, OC, menopausal status, age at menarche, age at menopause, age at first birth.
Cea-Soriano,2012	6	1996–2008	12–89	NCC	549/7347	Self-administered questionnaire or In-person interview	Age, index year, number of primary care physician visits.	HRT, OC,
Jhawar, 2003	1	1976–1996	30–55	Cohort	125/1,213,522	Self-administered questionnaire	Age, BMI, menopausal status, PMH use	HRT, OC, age at menarche, age at first birth, parity.
Benson, 2008	6	1996–2005/6.2	50–65	Cohort	390/124,967	Self-administered questionnaire	Age, height, BMI, strenuous exercise, socioeconomic level, smoking, alcohol intake	OC, age at first birth, parity.
Benson,2010	6	1996–2005/5.3	50–56	Cohort	311/1,147,894	Self-administered questionnaire	Age, socioeconomic status, residential area, height, BMI	HRT.
Michaud,2010	2,3,5,6,10, 11,12,13,14	1992–2008/8.4	20–83	Cohort	194/276,212	Self-administered questionnaire	Age, smoking status, education, BMI, menopausal status.	HRT, OC, menopausal status, age at menarche, age at menopause, age at first birth, parity.
Johnson,2011	1	1986–2004/10.5	55–85.7	Cohort	125/291,021	Self-administered questionnaire	Age	HRT, OC, age at menarche, age at menopause, age at first birth, parity.
Blitshteyn,2008	1	1993–2003	26–86	Cohort	1390/355,318	Medical records	Age	HRT
Andersen,2013	3	2000–2009	55–84	PCC	924/6122	Prescription Registry	Years of schooling, histories of diabetes, stroke, allergy or asthma and use of anti-asthma drugs and antihistamines	HRT

PCC, population-based case-control study; HCC, hospital-based case-control study; NCC, nested case-control study; BIM, body mass index.

^a^ Studies were conducted in: (1) USA, (2) Sweden, (3) Denmark, (4) Finland, (5) Norway, (6) the United Kingdom, (7) Australia, (8) France, (9) Canada, (10)Spain, (11)Italy, (12)Germany, (13)Greece, and (14)the Netherlands.

### Meta-analysis results

#### HRT use

**Figure 2:**
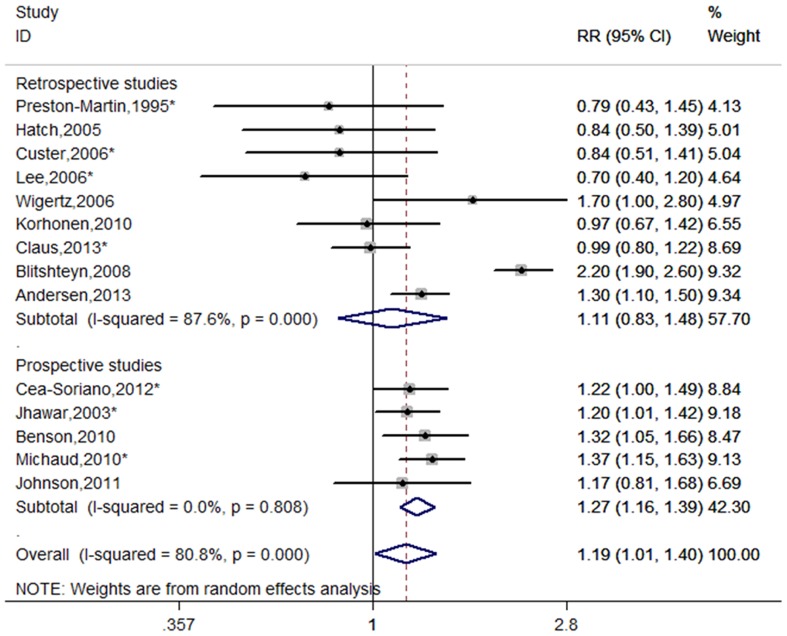
Forest plot of HRT use and meningioma risk. *The risk estimates are computed from raw data or abstracted from original studies.

Fourteen studies were included the meta-analysis [13], [16]–[20], [23]–[28], [33], [46]. Figure 2 shows the RRs of meningioma and HRT use, overall and by study design. The combined risk estimates were 1.11 (95%CI = 0.83–1.48, p for heterogeneity <0.001, I^2^ = 87.6%) for retrospective studies and 1.27 (95% CI = 1.16–1.39, p for heterogeneity  = 0.808, I^2^ = 0.0%) for prospective studies. Combining the retrospective and prospective data, the pooled risk estimate was 1.19 (95%CI = 1.01–1.40, p for heterogeneity <0.001, I^2^ = 80.8%). When subgroup analyses were conducted according to geographic regions, significant association were observed for European countries (RR = 1.29, 95% CI = 1.18–1.41, p for heterogeneity  = 0.532, I^2^ = 0.0%), but not for North America (RR = 1.07, 95% CI = 0.78–1.46, p for heterogeneity <0.001, I^2^ = 88.9%).

#### OC use

**Figure 3:**
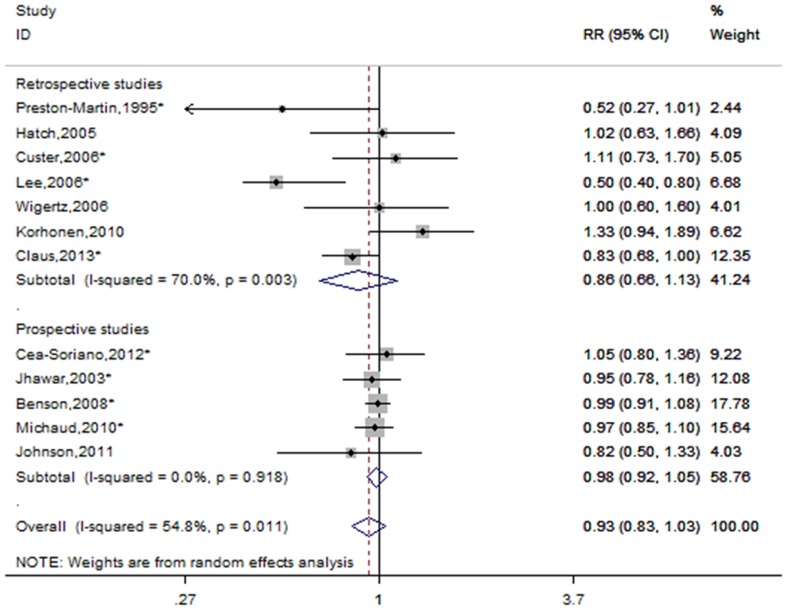
Forest plot of OC use and meningioma risk. *The risk estimates are computed from raw data or abstracted from original studies.

A total of twelve studies including seven retrospective studies and five prospective studies reported the risk estimates forever versus never OC use [13], [16]–[20], [21], [24]–[28]. Figure 3 shows the forest plots forever versus never OC use, overall and by study design. The cumulative estimated risks associated with ever OC use were 0.93(95% CI = 0.83–1.03, p for heterogeneity  = 0.011, I^2^ = 54.8%). When subgroup analyses were performed according to study design, no significant link was found in retrospective (RR = 0.86, 95% CI = 0.66–1.13, p for heterogeneity  = 0.003, I^2^ = 70.0%) or prospective studies (RR = 0.98, 95% CI = 0.92–1.05, p for heterogeneity  = 0.918, I^2^ = 0.0%). When subgroup analyses were conducted according to geographic regions, a marginal significant correlation was observed in North America (RR = 0.81, 95%CI = 0.66–0.99, p for heterogeneity  = 0.025, I^2^ = 58.4%), but not in European countries (RR = 1.00, 95% CI = 0.93-1.07-1.30, p for heterogeneity  = 0.565, I^2^ = 0.0%).

#### Menopausal status

**Figure 4:**
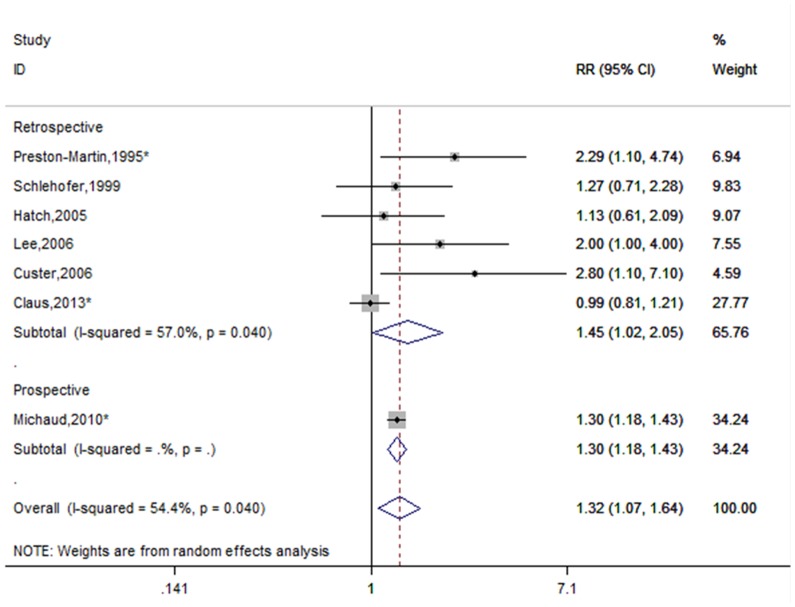
Forest plot of menopausal status and meningioma risk. *The risk estimates are computed from raw data or abstracted from original studies.

Seven studies analyzed the role of menopausal status on meningioma risk [13], [15], [17]–[19], [25], [28]. Figure 4presents the forest plots for meningioma incidence among postmenopausal women compared with premenopausal women. The summary RR was 1.32 (95%CI = 1.07–1.64, p for heterogeneity  = 0.040, I^2^ = 54.4%).

#### Age at menarche

**Figure 5:**
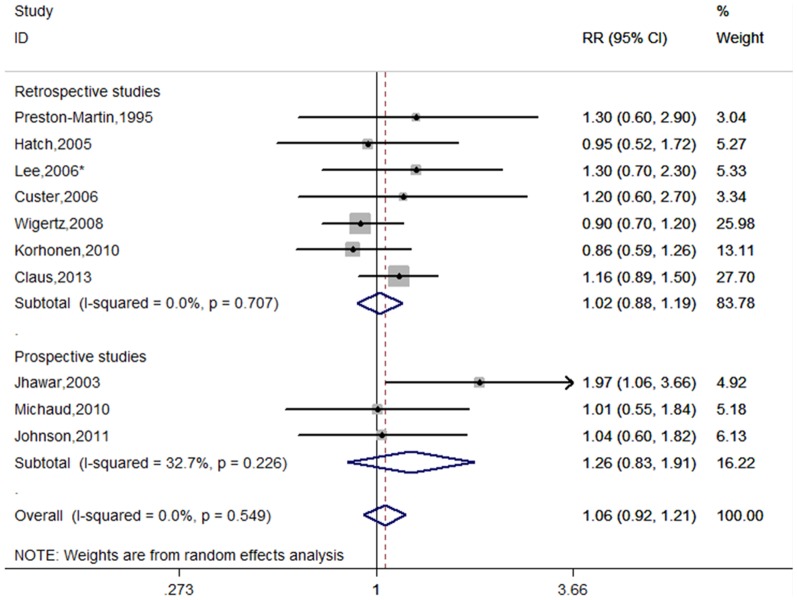
Forest plot of age at menarche and meningioma risk. *The risk estimates are computed from raw data or abstracted from original studies.

Associations of meningioma risk with age at menarche were reported in ten studies [13], [16]–[19], [22], [24]–[26], [28]. The pooled RR for the oldest age group (≥15 or 14 years) versus the youngest age group (≤11 or 12 years) was 1.06(95%CI = 0.92–1.21, p for heterogeneity  = 0.549, I^2^ = 0.0%), as is shown in Figure 5.

#### Age at menopause

**Figure 6:**
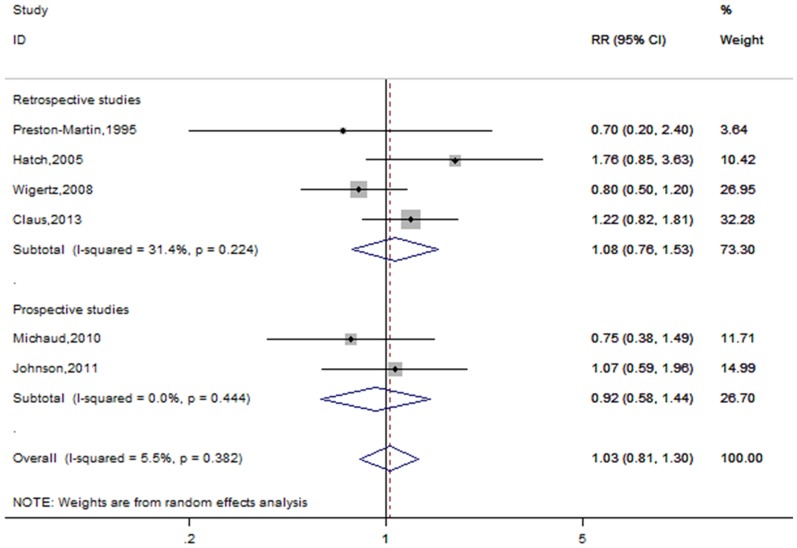
Forest plot of age at menopause and meningioma risk.

Risk estimates for oldest versus youngest age at menopause were reported in six studies [13], [17], [22], [25], [26], [28].The combined RR for the oldest age group (≥50 to ≥55 years) versus the youngest age group (≤40 to ≤47 years) was 1.03(95%CI = 0.81–1.30, p for heterogeneity  = 0.382, I^2^ = 5.5%), as is shown in Figure 6.

#### Age at first birth

**Figure 7:**
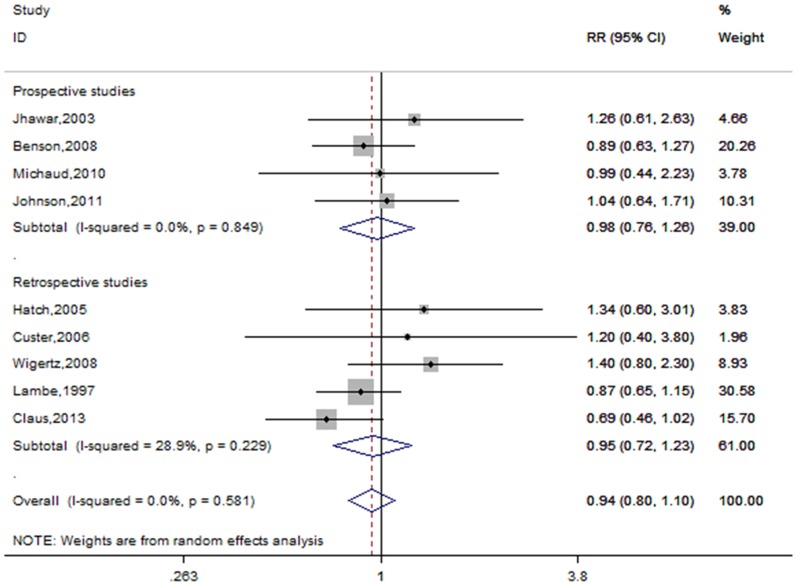
Forest plot of age at first birth and meningioma risk.

Nine studies examined the relationship between meningioma risk and age at first birth [14], [16], [17], [19], [21], [22], [25], [26], [28]. Figure 7 shows the forest plots for the oldest age group (≥20 to ≥23 years) versus the youngest age group (≥25 to ≥35 years). The pooled RR was 0.94(95%CI = 0.80–1.10, p for heterogeneity  = 0.581, I^2^ = 0.0%). Of 9 studies, two studies used the nulliparous women as the reference group [17], [25], whereas the others used parous women as reference group. Excluding these two studies [17], [25], the result was not significantly altered (RR = 0.92, 95% CI = 0.78–1.09, for heterogeneity  = 0.448, I^2^ = 0.0%).

#### Parity

**Figure 8:**
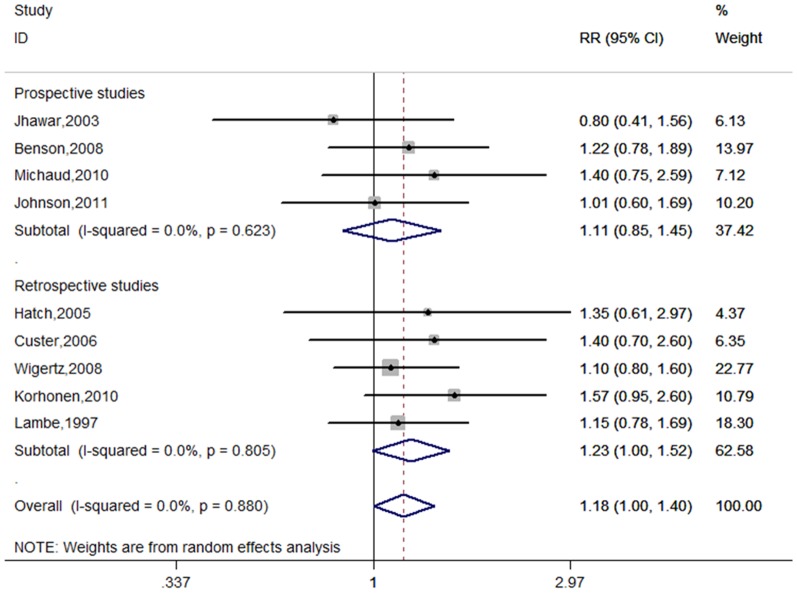
Forest plot of parity and meningioma risk.

Nine studies provided information on parity [14], [16], [17], [19], [21], [22], [24]–[26]. Figure 8 shows the forest plots for highest number of live births in comparison with the lowest. The summary RR was 1.18(95%CI = 1.00–1.40, p for heterogeneity  = 0.880, I^2^ = 0.0%). Among these studies, most studies used the nulliparous women as the reference group, whereas two studies used parous women as the reference group [16], [26]. Excluding the two studies, a similar result was observed (RR = 1.24, 95% CI = 1.04–1.49, p for heterogeneity  = 0.939, I^2^ = 0.0%).

### Sensitivity analysis

In the sensitivity analysis, we excluded one single study in turn to investigate the influence of a single study on the overall risk estimate. For HRT, no significant or marginal significant correlations were detected after excluding six studies [16], [20], [23], [25], [27], [46], as is shown in Table S1. For parity, no significant or marginal significant associations were observed after excluding six studies [14], [17], [19], [21], [24], [25], as is shown in Table S2. For other risk factors, the outcomes were not significantly altered (data not shown).

We also performed a sensitivity analysis restricted to those studies that provided adjusted risk estimates. The combined RRs for menopause status, age at menarche, HRT use, and OC use were 1.53 (95% CI = 1.06–2.21, p for heterogeneity  = 0.320, I^2^ = 14.4%), 1.04 (95% CI = 0.91–1.20, p for heterogeneity  = 0.498, I^2^ = 0.0%), 1.33 (95% CI = 1.02–1.72, p for heterogeneity <0.001, I^2^ = 84.8%), and 1.08 (95% CI = 0.87–1.34, p for heterogeneity  = 0.435, I^2^ = 0.0%), respectively.

### Publication bias

The results of Egger' test suggested there was no evidence of notable publication bias (p = 0.376 for OC; p = 0.057 for HRT; p = 0.410 for menopausal status; p = 0.245 for age at menarche; p = 0.841 for age at menopause; p = 0.079 for age at first birth; p = 0.662 for parity). For HRT, we further employed “trim and fill” method [47], but this analysis suggested the result was unchanged.

## Discussion

This meta-analysis included six cohort and twelve case-control studies to evaluate the relationship between OC use, HRT use, reproductive factors and risk of meningioma. Our analysis confirmed that OC use, age at menarche, age at menopause, and age at first birth did not significantly contribute to the risk of developing meningioma. However, this meta-analysis showed that HRT use, postmenopausal status and increasing number of births were associated with an increased risk of meningioma.

Our meta-analysis suggested HRT use was correlated with an elevated risk of meningioma in women. This finding is consisted with the newly published meta-analysis, which included six case-control and five cohort studies [48]. Moreover, some interesting findings were shown in the newly published meta-analysis. Fan and colleagues found the significant risk elevation was observed in current users (RR = 1.27, 95% CI = 1.08–1.49), but not in past users (RR = 1.12, 95% CI = 0.95–1.32).The significance of these findings is unclear. However, we should interpret these findings with caution because meningiomas usually have an extended duration of asymptomatic subclinical disease before presentation to medical attention. Lastly, two important issues were not addressed in these two meta-analyses. One is that we were unable to take into account the type of OC and HRT. Several previous studies had suggested progesterone and estrogen receptors are expressed in meningioma in various degrees [6]–[7]. Recently, two studies have reported that women with estrogen/estradiol-alone therapy were associated with a slightly increased risk of meningioma, while this risk was not observed for the users of a combination of estrogen/estradiol and progestin [24], [45]. Another is that the dose-response analysis was not performed. Assessment of dose-response is considered to be a major criterion for determination of the causality for association in observational studies. Therefore, it is unknown whether the results were detected by chance or not. In order to determine which kind of hormones or to what extent hormone use influences the risk of meningioma, further evaluation of hormone use in women with meningioma is needed to pay more attention to stratification by hormone composition (i.e., estrogen and/or progesterone), duration of use, dosage of use, and age at start/end of therapy as well as tumor receptor subtype.

In the current study, an increased risk of meningioma was observed among postmenopausal women in comparison with premenopausal women. Our finding seems to be conflicted with the hypothesis that female sex hormones have a promoting effect on meningioma incidence. This could be explained because most studies did not take into account the effect of HRT and length of exposure [49]. Among included studies, only two studies provided a detailed description on definition of postmenopausal status [26], [28]. However, both studies reported that women who implied use of exogenous hormones while still menstruating were also defined as postmenopausal. Thus, it seems difficult here to assess the role of menopause in the incidence of meningioma independently of HRT use. Furthermore, some authors may not take into account exposure occurring shortly prior to the reference date, since it was assumed that it was unlikely to play a role in the disease. Lastly, this finding that meningioma occurred more commonly in postmenopausal women may be due to the bias. Since many meningiomas are asymptomatic, they can be present starting in a younger age and be discovered in an older age. Moreover, older adults tend to have more diagnostic testing for health problems, for example a slip and fall and cardiovascular and cerebrovascular disease, which are completely unrelated to the meningioma [50]. We would therefore see more and more older people with meningioma. Therefore, our finding should be interpreted with caution and further evaluation of menopausal status should take into account the date of exposure occurring before the date of diagnosis (or date of interview for controls) and HRT use.

With regard to other reproductive factors, we conducted a meta-analysis for comparison of the highest versus lowest category in each study. No significant correlations were observed for age at menarche, age at menopause or age at first birth. In contrast, an elevated risk of meningioma was found with parity, which is consistent with biological hypothesis. Women with greater numbers of live births or full-term pregnancies would be under a longer period of exposure to high levels of progesterone and estrogen. Thus, these women may bear a larger risk of hormone-induced meningioma than those with fewer numbers of live births or full-term pregnancies. However, the result should also be interpreted with caution because our sensitivity analysis showed the results were not robust.

Finding from this meta-analysis showed that female sex hormones play a role in the risk of meningiomas in women, but there is also a study that suggested a hormonal influence on meningiomas in men [51]. Aghi et al found that male patients with meningiomas exhibited a higher average body mass index (BMI) and higher obesity rate in comparison with male patients with aneurysms or gliomas and that obese male patients with meningiomas presented higher rates of postoperative complications (postoperative deep vein thrombosis, pulmonary embolus, and fever) than nonobese male patients with meningiomas [51]. Obesity has been shown to increase serum estradiol and insulin-like growth factor (IGF), which, in turn, link obesity to carcinogenesis [52], [53]. Furthermore, epidemiological evidence suggested obesity increases the risk of several hormone-dependent neoplasms (i.e., endometrial, breast, uterine, ovarian, and prostate cancers) in both men and women [53], [54]. Since meningionas are known to be hormonally sensitive tumors, it would not be surprising that hormones also have an effect on meningiomas in men.

Several biological mechanisms explaining how female hormones could possibly increase the risk of hormone-related cancers have been proposed. The female hormones can modulate proliferation and cell cycle progression through transcriptional mechanisms involving the receptors [8], [20]. In addition, estrogens have been postulated to affect the genomic instability of cells [55], [56]. Lastly, estrogens interact with IGF, which stimulates tumor growth and prohibits cells apoptosis [52].

Some limitations of the current study should be considered when interpreting our results. First, this study was limited by the retrospective data and lack of sufficient prospective evidence. The existing recall and selection bias would confound the association between hormone and reproductive factors and risk of meningioma. Most of the studies (n = 12) included in this study were retrospective studies. In the retrospective studies of meningioma, the recall bias may be even greater because patients are often experiencing effects of cerebral lesions and surgery, which affect their cognition or memories. Furthermore, estimation of hormone use or reproductive factors in most studies was through the self-reported and proxy-reported measures. Both methods of assessing the exposure would contribute to recall bias and measurement error. Second, substantial heterogeneity across studies was observed. Finding the source of heterogeneity is often a concern in a meta-analysis. In our study, the heterogeneity contained the following several aspects: (i) the study designs were different. Twelve retrospective and six prospective studies were included in this study. (ii) Studies included in this study were conducted in different geographic regions: either entirely in Europe or entirely in North America, where people share little in the field of genetic background and lifestyle. (iii) Both spinal and intracranial meningiomas were included in this study. Though spinal and intracranial meningiomas arise from meningothelial cells of the arachnoid membrane, spinal meningioma is less common than intracranial meningioma [2]. This may suggest that the etiology of tumors is different, which may, in part, explain some heterogeneity. (iv)The studies used different methods to collect information. Assessment tools to get information of exposure variables consisted of in person interview, telephone interview, self-administered questionnaire, and reviewing medical records. With different methods, the participants may have different attitudes towards the questions. Consequently, the reliability of the answers to question about exposures might be questionable. Third, unmeasured and residual confounders from original studies are always of concern in observational studies. Most risk estimates were derived from multivariable models, but the adjusted factors in each study were different. Therefore, we could not preclude the possibility that other unmeasured or inadequately measured factors have confounded the relationship. Fourth, potential publication bias might influence our findings. Egger's test suggested that no evidence of publication bias was observed in the present meta-analysis, but we cannot exclude the possibility that some unpublished studies may have been missed during our literature search, and that studies with null effects tend to be unpublished. Fifth, some meningiomas were diagnosed on radiological image, without histopathological confirmation. This may contribute to some unclear bias because some meningiomas diagnosed on radiological criteria may be completely independent of the pathogenesis which was proposed to be related to hormonal levels. Finally, ethnic differences could play an important role in the development of neoplasms. In this study, we found that all of the studies involved Western populations. Therefore, additional research in other populations is warranted to extend the findings.

In summary, we found an elevated risk of female meningioma with HRT use, postmenopausal status and parity, which is consistent with the hypothesis that female sex hormones could modulate the risk of meningioma in women. Further studies are warranted to extend this finding and clarify the underlying mechanisms.

## Supporting Information

Checklist S1 **PRISMA Checklist for this meta-analysis.**
(DOC)

Table S1 Results of sensitivity analysis for HRT.
(DOCX)

Table S2 Results of sensitivity analysis for parity.
(DOCX)
