# Supplementary material for: Reproductive and Exogenous Hormone Factors in Relation to Risk of Meningioma in Women: A Meta-Analysis
Source: PLoS One. 2013 Dec 27;8(12):e83261. doi: 10.1371/journal.pone.0083261 (PMC3873952; doi:10.1371/journal.pone.0083261)
Supplement: Table S1 — Results of sensitivity analysis for HRT. (DOCX) [file pone.0083261.s002.docx]

Table S1 Results of sensitivity analysis for HTR use and meningioma risk.

| Study omitted | RR(95% CI) |
| --- | --- |
| Preston-Martin,1995 | 1.21 (1.03-1.43) |
| Hatch,2005 | 1.21 (1.03-1.43) |
| Custer,2006 | 1.21 (1.03-1.43) |
| Lee,2006 | 1.22 (1.04-1.44) |
| Wigertz,2006 | 1.17 (0.99-1.38) |
| Korhonen,2010 | 1.21 (1.02-1.43) |
| Cea-Soriano,2012 | 1.18 (0.99-1.41) |
| Claus,2013 | 1.21 (1.03-1.43) |
| Jhawar,2003 | 1.18 (0.99-1.41) |
| Benson,2010 | 1.18 (0.99-1.40) |
| Michaud,2010 | 1.17 (0.98-1.40) |
| Johnson,2011 | 1.19 (1.01-1.41) |
| Blitshteyn,2008 | 1.17 (1.06-1.28) |
| Andersen,2013 | 1.17 (0.98-1.41) |
| All studies | 1.19 (1.01-1.40) |
