# Supplementary material for: Reproductive and Exogenous Hormone Factors in Relation to Risk of Meningioma in Women: A Meta-Analysis
Source: PLoS One. 2013 Dec 27;8(12):e83261. doi: 10.1371/journal.pone.0083261 (PMC3873952; doi:10.1371/journal.pone.0083261)
Supplement: Table S2 — Results of sensitivity analysis for parity. (DOCX) [file pone.0083261.s003.docx]

Table S2 Results of sensitivity analysis for parity and meningioma risk.

| Study omitted | RR(95% CI) |
| --- | --- |
| Jhawar,2003 | 1.22 (1.02-1.44) |
| Hatch,2005 | 1.18 (0.99-1.39) |
| Custer,2006 | 1.17 (0.99-1.39) |
| Wigertz,2008 | 1.21 (1.00-1.46) |
| Benson,2008 | 1.18 (0.99-1.41) |
| Korhonen,2010 | 1.15 (0.96-1.36) |
| Michaud,2010 | 1.17 (0.99-1.39) |
| Johnson,2011 | 1.21 (1.01-1.44) |
| Lambe,1997 | 1.19 (0.99-1.43) |
| All studies | 1.18 (1.00-1.40) |
